# The contribution of travellers visiting friends and relatives to notified infectious diseases in Australia: state-based enhanced surveillance

**DOI:** 10.1017/S0950268816001734

**Published:** 2016-08-30

**Authors:** A. E. HEYWOOD, N. ZWAR, B. L. FORSSMAN, H. SEALE, N. STEPHENS, J. MUSTO, C. LANE, B. POLKINGHORNE, M. SHEIKH, M. SMITH, H. WORTH, C. R. MACINTYRE

**Affiliations:** 1 School of Public Health & Community Medicine, UNSW Australia, Sydney, NSW, Australia; 2 Public Health Unit, Nepean Blue Mountains Local Health District, NSW, Australia; 3 Health Protection Branch, Victorian Department of Health and Human Services, Melbourne, Victoria, Australia; 4 NSW Ministry of Health, Sydney, NSW, Australia; 5 NSW Refugee Health Service, NSW, Australia

**Keywords:** Australia, enhanced surveillance, immigrants, infectious diseases, travel, visiting friends and relatives

## Abstract

Immigrants and their children who return to their country of origin to visit friends and relatives (VFR) are at increased risk of acquiring infectious diseases compared to other travellers. VFR travel is an important disease control issue, as one quarter of Australia's population are foreign-born and one quarter of departing Australian international travellers are visiting friends and relatives. We conducted a 1-year prospective enhanced surveillance study in New South Wales and Victoria, Australia to determine the contribution of VFR travel to notifiable diseases associated with travel, including typhoid, paratyphoid, measles, hepatitis A, hepatitis E, malaria and chikungunya. Additional data on characteristics of international travel were collected. Recent international travel was reported by 180/222 (81%) enhanced surveillance cases, including all malaria, chikungunya and paratyphoid cases. The majority of cases who acquired infections during travel were immigrant Australians (96, 53%) or their Australian-born children (43, 24%). VFR travel was reported by 117 (65%) travel-associated cases, highest for typhoid (31/32, 97%). Cases of children (aged <18 years) (86%) were more frequently VFR travellers compared to adult travellers (57%, *P* < 0·001). VFR travel is an important contributor to imported disease in Australia. Communicable disease control strategies targeting these travellers, such as targeted health promotion, are likely to impact importation of these travel-related infections.

## INTRODUCTION

Travel is a major pathway for the spread of infections globally, including diseases which constitute a public health emergency of international concern, and international travellers are an important source of infectious diseases in countries with robust national disease control systems and low disease incidence, such as Australia. When travelling from developed to less developed countries, the risk of infectious disease is greater in those travelling to visit friends and relatives (VFR) in their country of origin compared to those travelling for other purposes [1, 2]. Of returning travellers presenting at clinics affiliated with the GeoSentinel surveillance system, a higher proportion of VFR travellers present with serious, potentially preventable travel-related illnesses than travellers for other purposes [1]. VFR travellers are more likely to stay for extended periods of time [1, 3], thus increasing opportunities for exposure. Exposure may also be increased through closer contact with the local population, consumption of local food and water supply, and poor adherence to mosquito avoidance practices [2, 4, 5], placing VFR travellers at increased risk of respiratory, vector-borne and food- and water-borne illnesses. Compared to other travellers, VFR travellers are less likely to seek pre-travel health advice from a health professional [4, 6], less likely to be vaccinated [7] or take malaria chemoprophylaxis [8]. Reasons for low uptake of pre-travel health advice are multifactorial and relate to access, lack of awareness of the need for advice and low-risk perception [2, 8].

Globally, VFR travellers comprise about one-quarter of international travel episodes and this is forecast to increase [9]. Of the 9·1 million short-term international departures by Australian residents in 2014, 23% were for the purposes of VFR [10], although this proportion can reach up to 46% for travel to South and Central Asia [11]. Despite a high volume of international travel to and from Australia, detailed information on the contribution of travel to notifiable diseases is poorly captured in national data [12] and reason for travel is not routinely collected. Notifiable disease data does indicate a high proportion of travel-associated disease in immigrant Australians, including hepatitis A [13, 14], hepatitis E [13], typhoid [13], paratyphoid [13], tuberculosis [15, 16] and malaria [17]. While routine surveillance data does include country of birth, other indicators of ethnicity such as parents’ country of birth and language spoken at home are not reporting requirements, thereby not capturing disease in Australian-born children of immigrants. We aimed to determine the contribution of VFR travel to travel-associated notifiable diseases in Australia's two most populous states: New South Wales (NSW) and Victoria.

## METHODS

Prospective enhanced surveillance of newly notified cases of typhoid, paratyphoid, measles, hepatitis A, hepatitis E, chikungunya and malaria was conducted in the states of NSW and Victoria from February 2013 to January 2014, inclusive. NSW and Victoria comprise 57% of the Australian population [18] and 65% of resident Australian travellers depart from airports in these states [19]. These infectious diseases were included as they are commonly acquired during travel, have little to no local spread within Australia, are also preventable (except for hepatitis E and paratyphoid) by vaccination or through mosquito avoidance measures and are therefore of interest in terms of disease prevention in travellers, they have good case ascertainment and are actively followed up by public health units.

Australia has a national notifiable disease surveillance system (NNDSS). Surveillance is the responsibility of each State and Territory and standard national case definitions [20] are used to confirm cases that are reportable to the NNDSS. In each jurisdiction, legislation requires doctors and/or laboratories to notify over 50 diseases [21]. Data collected by State and Territory public health officers includes the following demographic characteristics: country of birth, disease severity (hospitalization, death), and limited data on risk factors for disease acquisition, including recent travel [22]. Data are not collected on reason for travel or ethnicity. In NSW and Victoria, notification of hepatitis A, hepatitis E, typhoid, paratyphoid and measles requires public health action with follow-up of all cases by public health officers. In Victoria, notifiable disease surveillance is centralized, whereas, in NSW follow-up of notified cases is conducted by regional public health units and then reported centrally. Notification of malaria and chikungunya requires public health action only when there is a risk of local transmission as determined by the treating physician. However, routine follow-up of all reported chikungunya cases was undertaken during the data collection period in Victoria.

Eligible participants were confirmed cases of the selected diseases in Australian residents, including temporary residents, notified to the included jurisdictions during the study period. Arriving immigrants and overseas temporary visitors were excluded as their travel plans were not made in Australia and contact details of temporary visitors were not available. During routine follow-up of notified cases of hepatitis A, hepatitis E, typhoid, paratyphoid and measles (and chikungunya for Victoria only), public health surveillance officers sought permission from the case, or the case's parent/guardian, for study investigators to contact them by telephone to administer an enhanced surveillance questionnaire. On notification of malaria (and chikungunya in NSW), in which routine follow-up is not performed, public health surveillance officers provided details of the notifying medical practitioner to study investigators, who then sought permission to contact their patient.

Study investigators contacted participants or the parent/guardian of cases aged <18 years. Verbal informed consent was obtained from participants prior to administering the questionnaire. The enhanced surveillance questionnaire included details of any international travel within the incubation period, reason for travel and length of stay. Reason for travel was collected as a self-reported response as well as a list of possible other reasons for travel. For the purposes of this study, Australian residents born outside Australia were termed immigrants. If one or both parents were born outside Australia, the participants were termed Australian-born children of immigrants. We defined a ‘VFR traveller’ as those participants who self-reported visiting friends or relatives as a reason for travel, as per the capture of reason for travel on immigration overseas arrival and departure cards [10]. Geographical region of birth and travel destination were classified using the United Nations classification [23]. Data on all notified cases in the jurisdictions during the study period was obtained, by age, gender and country of birth to determine response rates and sample representativeness. Data were analysed using SPSS Statistics v. 22.0. (IBM Corp., USA). Statistical associations between categorical variables were analysed using *χ*^2^ test and linear variables were analysed using independent sample *t* test for differences in mean age or Mann–Whitney *U* and Kruskal–Wallis tests for differences in median trip duration. *P* values of <0·05 were considered significant. This study was approved by the NSW Population & Health Services Research Ethics Committee (2012/04/382).

## RESULTS

### Notifications and fieldwork results

Between February 2013 and January 2014, 634 confirmed cases of the included diseases were notified to NSW Health and the Victorian Department of Health. Of notified cases, 194 (30·6%) were aged 0–19 years, and 285 (45·0%) were female. Of the notified cases, 220 (34·7%) were Australian-born, 297 (46·8%) were born in a country other than Australia and 117 (18·5%) had no country of birth recorded. Of the overseas-born notified cases, 108 (36·4%) were Indian-born, the most common country of birth after Australia for all included diseases except measles (Table 1). Of the 527 (83·1%) notifications with contact details available, 21 (4·0%) were arriving immigrants and 35 (6·6%) were overseas visitors to Australia and ineligible for interview. Overall, 222 cases participated in the enhanced surveillance, 54·5% of those for whom contact details were available and 35·0% of total notifications. There was no significant difference between total notified cases and enhanced surveillance cases by 10-year age group (*P* = 0·34) or gender (*P* = 0·90). While a similar proportion were immigrants (*P* = 0·9), data were missing on country of birth for 117 notified cases (18·5%).

**Table 1.** Demographic characteristics of total notified cases (N = 634) and included enhanced surveillance cases (N = 222)

	Typhoid	Paratyphoid	Measles	HAV	HEV	Malaria	Chikungunya	Total
Demographics	*N* (%)	*N* (%)	*N* (%)	*N* (%)	*N* (%)	*N* (%)	*N* (%)	*N* (%)
**Notified cases**	97	48	106	122	24	182*	55	634
Age (years)								
0–19	43 (44)	9 (19)	56 (53)	52 (43)	4 (17)	28 (15)	2 (4)	194 (30·6)
20–39	38 (39)	29 (60)	40 (38)	44 (36)	13 (54)	86 (47)	14 (26)	264 (41·6)
40–59	15 (16)	7 (15)	10 (9)	16 (13)	4 (17)	47 (26)	29 (53)	128 (20·2)
⩾60	1 (1)	3 (6)	0	10 (8)	3 (13)	20 (11)	10 (18)	47 (7·4)
Sex (female)	52 (54)	31 (65)	45 (43)	53 (43)	8 (33)	59 (32)	37 (67)	285 (45·0)
Country/region of birth								
Australia	24 (25)	18 (38)	72 (68)	59 (48)	7 (29)	20 (11)	20 (36)	220 (34·7)
India	41 (42)	14 (29)	0	13 (11)	7 (29)	28 (15)	5 (9)	108 (17·0)
Other South Asia	14 (14)	4 (8)	2 (2)	13 (11)	3 (13)	6 (3)	0	42 (6·6)
South East Asia	7 (7)	5 (10)	12 (11)	10 (8)	3 (13)	6 (3)	1 (2)	44 (6·9)
Other	8 (8)	3 (6)	14 (13)	17 (14)	3 (13)	55 (30)	3 (6)	103 (16·2)
Not specified	3 (3)	4 (8)	6 (6)	10 (8)	1 (4)	67 (37)	26 (47)	117 (18·5)
**Enhanced surveillance cases**	35 (36)	25 (52)	44 (42)	58 (48)	14 (58)	26 (14)	20 (36)	222 (35·0)
Age (years)								
0–19	18 (51)	6 (24)	23 (52)	31 (53)	3 (21)	2 (8)	0	83 (37·4)
20–39	13 (37)	15 (60)	16 (36)	16 (28)	7 (50)	14 (54)	2 (10)	83 (37·4)
40–59	4 (11)	2 (8)	5 (11)	9 (16)	2 (14)	8 (31)	12 (60)	42 (18·9)
⩾60	0	2 (8)	0	2 (3)	2 (14)	2 (8)	6 (30)	14 (6·3)
Sex (female)	20 (43)	15 (40)	16 (64)	24 (59)	5 (64)	6 (77)	13 (35)	99 (55·4)
University education†	14 (78)	15 (79)	10 (43)	20 (69)	5 (42)	12 (52)	10 (53)	86 (60·1)
Immigrant status:								
Australian-born, Australian-born parents	2 (6)	6 (24)	17 (37)	14 (24)	1 (7)	5 (19)	10 (50)	55 (24·8)
Australia-born, immigrant parents	11 (31)	3 (12)	14 (32)	26 (45)	3 (21)	3 (12)	2 (10)	62 (27·9)
Immigrant	22 (63)	16 (64)	13 (30)	18 (31)	10 (71)	18 (69)	8 (40)	105 (47·3)
Length of immigration ⩽5 years‡	7 (33)	3 (19)	3 (23)	7 (39)	1 (10)	4 (22)	0	25 (24·3)
Length of immigration ⩽10 years‡	18 (86)	12 (75)	5 (39)	14 (78)	6 (60)	10 (56)	1 (14)	66 (64·1)
Speaks a language other than English at home	32 (91)	17 (68)	17 (39)	39 (67)	12 (86)	16 (62)	7 (37)	140 (63·3)
Region of birth								
Australia	13 (37)	9 (36)	31 (71)	40 (69)	4 (29)	8 (31)	12 (60)	117 (52·7)
South and Central Asia	18 (51)	12 (48)	0	8 (14)	6 (43)	3 (12)	4 (20)	51 (23·0)
South East Asia	1 (3)	3 (12)	7 (16)	2 (3)	3 (21)	1 (4)	2 (10)	19 (8·6)
Middle East	0	0	1 (2)	2 (3)	0	0	0	3(1·4)
Pacific (including New Zealand)	3 (9)	1 (4)	1 (2)	4 (7)	0	3 (12)	0	12 (5·4)
Sub-Saharan Africa	0	0	0	0	0	9 (35)	0	9 (4·1)
Europe	0	0	4 (9)	1 (2)	1 (7)	2 (8)	2 (10)	10 (4·5)
North America	0	0	0	1 (2)	0	0	0	1 (0·5)
Parents region of birth§								
Australian	0	0	6 (43)	6 (23)	1 (33)	1 (33)	0	14 (22·6)
South and Central Asia	10 (91)	3 (100)	3 (21)	4 (15)	2 (67)	0	0	22 (35·5)
South-East Asia	0	0	4 (29)	2 (8)	0	0	0	6 (9·7)
Middle East	0	0	1 (7)	16 (62)	0	0	0	17 (27·4)
Pacific (including New Zealand)	1 (9)	0	1 (7)	0	0	1 (33)	0	3 (4·8)
Sub-Saharan Africa	1 (9)	0	0	0	0	2 (68)	0	3 (4·8)
Europe	0	0	6 (43)	3 (12)	1 (33)	0	1 (100)	11 (17·7)
North America	0	0	0	2 (8)	0	0	0	2 (3·2)

HAV, Hepatitis A virus; HEV, hepatitis E virus.

* Includes one case in which age was not recorded.

† Adult cases only, *n* = 143.

‡ Migrants only, *n* = 105.

§ Australian-born with migrant parents only, *n* = 62 (multiple response allowed).

### Demographic characteristics

Of the 222 participants who completed the enhanced surveillance, 99 (45%) were female, with a median age of 26·5 years (range 0–80 years), including 77 (35%) children aged <18 years. The proportion of children aged <18 years was highest for hepatitis A (29, 50%), typhoid (17, 49%) and measles (21, 48%). Only two cases of malaria (8%) and no cases of chikungunya occurred in children. The mean age of cases of vaccine-preventable diseases (hepatitis A, measles, typhoid) was lower (21·7 years, s.d. = 16·2) compared to other diseases (37·7 years, s.d. = 18·9, *t* = 6·700, d.f. = 220, *P* < 0·001).

Overall, 205 (92·3%) cases were Australian citizens or permanent residents, six were international students, nine temporary residents (work or family visas) and four New Zealand citizens residing in Australia. One hundred and five (47·3%) cases were immigrants, with 89 (85%) originating from low- or middle-income countries, including 40 (38%) born in India. No other country of birth contributed more than seven cases. Of the immigrant cases, 25 (24%) had migrated to Australia <5 years previously, with 66 (64%) migrating within the last 10 years. Of the 117 Australian-born cases, 62 (53%) had immigrant parents, including 14 (23%) with one parent born in Australia and 49 (79%) with at least one parent originating from a low- or middle-income country. Lebanon (11, 18%) and Pakistan (10, 16%) were the most common countries of parents’ birth of these Australian-born cases. Demographic characteristics by disease are shown in Table 1.

### Recent travel-history

A history of recent international travel was reported by 180 (81%) cases, including all cases of malaria, chikungunya and paratyphoid. There were three cases of typhoid in people with no recent travel – two carriers, and one secondary case from a carrier. Of the 19 cases of hepatitis A acquired in Australia, seven (37%) were contacts of an Australian-born child with immigrant parents. Immigrant cases (96, 91%) were more likely to have a history of recent travel than Australian-born cases (84, 72%, *P* < 0·001). Travel histories by disease are shown in Table 2.

**Table 2.** Travel characteristics of enhanced surveillance cases reporting recent travel (N = 180)

	Typhoid	Paratyphoid	Measles	HAV	HEV	Malaria	Chikungunya	Total
Trip characteristic	*N* (%)	*N* (%)	*N* (%)	*N* (%)	*N* (%)	*N* (%)	*N* (%)	*N* (%)
Recent travel	32/35 (91)	25/25 (100)	25/44 (57)	39/58 (67)	13/14 (93)	26/26 (100)	20/20 (100)	180/222 (81·1)
Reason for travel*								
Holiday	12 (38)	12 (48)	19 (76)	29 (74)	7 (54)	7 (27)	14 (70)	100 (55·6)
VFR	31 (97)	18 (72)	11 (44)	24 (62)	10 (77)	14 (54)	9 (45)	117 (65·0)
Business/study	1 (3)	2 (8)	3 (12)	2 (5)	1 (8)	10 (39)	2 (10)	21 (11·7)
Other†	3 (9)	3 (12)	0	0	3 (23)	3 (12)	2 (10)	14 (7·8)
Travel to COB/parents COB	28 (88)	17 (68)	12 (48)	22 (56)	8 (62)	15 (58)	6 (30)	108 (60·0)
Length of travel								
1 to <2 weeks	0	3 (12)	7 (28)	4 (10)	0	1 (4)	9 (45)	24 (13·3)
2 weeks to <1month	8 (25)	7 (28)	13 (52)	14 (36)	3 (23)	4 (15)	6 (30)	55 (30·6)
1 to <2months	16 (50)	9 (36)	2 (8)	10 (26)	7 (54)	10 (39)	3 (15)	57 (31·7)
2 to <3months	8 (25)	4 (16)	1 (4)	10 (26)	3 (23)	2 (8)	1 (5)	29 (16·1)
⩾3 months	0	2 (8)	2 (8)	1 (3)	0	9 (35)	1 (5)	15 (8·3)
Travel region*								
South/Central Asia	30 (94)	18 (72)	3 (12)	15 (39)	10 (77)	4 (15)	5 (25)	85 (47·2)
South East Asia	3 (9)	11 (44)	13 (52)	12 (31)	7 (54)	6 (23)	15 (75)	72 (40·0)
North Asia	0	0	0	2 (5)	1 (8)	0	0	3 (1·7)
Middle-East and North Africa	0	0	1 (4)	7 (18)	2 (15)	3 (12)	1 (5)	14 (7·8)
Sub-Saharan Africa	0	0	0	1 (3)	0	21 (81)	0	22 (12·2)
Europe	0	0	3 (12)	5 (14)	1 (8)	0	0	9 (5·0)
Pacific	1 (3)	0	1 (4)	9 (23)	0	4 (15)	0	15 (8·3)
South/Central America	0	1 (4)	0	0	0	0	0	1 (0·6)

HAV, Hepatitis A virus; HEV, hepatitis E virus; VFR, visiting friends and relatives; COB, country of birth.

* Multiple reasons/regions allowed.

† Other includes: attending weddings, funerals and caring for sick relatives, pilgrimage, and medical treatment.

Of the 180 cases with a history of recent travel, 117 (65%) were travelling for the purpose of VFR. Reasons for travel by country of birth and parents’ country of birth are shown in Table 3. Multiple reasons for travel were reported by 72 (40%) travel-associated cases, 68 (94%) of which included VFR travel. Fifty-three (45%) out of 117 VFR travellers also reported holiday travel as their travel purpose. Travel to the case's country of birth was reported by 78/96 (81%) of immigrants. For Australian-born cases with immigrant parents, travel to their parents’ country of birth was reported by 29/43 (67%). Of the travel cases, 51 (28%) were in children aged <18 years. Children were more likely to be VFR travellers (44/51, 86%) compared to adult travel cases (73/129, 57%, *P* < 0·001). Of the paediatric cases, all hepatitis E, malaria and typhoid cases were VFR travellers and all but one hepatitis A and paratyphoid cases.

**Table 3.** Reason for travel by immigrant status of travel-associated enhanced surveillance cases (N = 180)

	Immigrant	Australian-born/immigrant parents	Australian born/Australian-born parents
	(*N* = 96)	(*N* = 43)	(*N* = 41)
Reason for travel*	*n* (%)	*n* (%)	*n* (%)
VFR	81 (84)	32 (74)	4 (10)
Holiday	42 (44)	25 (58)	33 (81)
Business/Study	9 (9)	4 (9)	8 (20)
Other†	12 (13)	1 (2)	1 (2)

VFR, Visiting friends and relatives.

* Multiple responses allowed.

† Other includes: weddings, funerals, pilgrimage, or medical treatment as per response. Weddings and funerals also classified as VFR travel

Those travelling to visit friends and relatives had significantly longer trip duration (median 36 days, *vs*. 16 days, *P* < 0·001) compared to those travelling for other purposes. Differences in trip duration by disease and migration status are shown in Table 4. Of those with recent travel, 143 (79%) reported travel to one country, 26 (11%) to two countries, and 11 (6%) to three or four countries. Overall, the most common countries visited by cases travelling for VFR purposes were India (52, 44%), Pakistan, (12, 10%), Thailand (10, 9%) and the Philippines (7, 6%). The most common travel destinations for other travellers were Indonesia (17, 27%), Thailand (9, 14%), Singapore (5, 8%) and Fiji (5, 8%), although all but one traveller to Singapore also travelled to other destinations. Regions visited by notified disease are shown in Table 2.

**Table 4.** Differences in length of trip by travel characteristics and immigrant status (N = 180)

Factor	Range (days)	Median	*P*
Typhoid	14–82	32	<0·001‡
Paratyphoid	6–365	34	
Measles	7–730	18	
HAV	4–104	32	
HEV	16–73	31	
Malaria	11–304	47·5	
Chikungunya	6–300	16·5	
VFR travellers	4–365	36	<0·001†
Other travellers	6–730	16	
Australian-born/Australian-born parents	6–290	15	<0·001‡
Australian-born/immigrant parents	4–365	31	
Immigrant Australians	7–730	32	

HAV, Hepatitis A virus; HEV, hepatitis E virus; VFR, visiting friends and relatives.

† Mann–Whitney *U* test.

‡ Kruskal–Wallis test.

## DISCUSSION

Our enhanced surveillance study of notifiable diseases shows that VFR travellers are the largest risk group for imported diseases in Australia. This has direct implications for targeting disease control strategies for infectious diseases, many of which may result in local outbreaks. VFR travellers are at increased risk of disease acquisition during travel and subsequent importation [1]. In this study, 65% of travel-associated cases were VFR travellers and 47% were in immigrant Australians, despite the fact that VFR travel comprises only 23% of departures by Australian residents [10] and a quarter of Australia's population are immigrants [24]. VFR travel by immigrants and their children is an important travel group for imported disease; in this study, it was a factor in the majority of cases of hepatitis A, hepatitis E, typhoid, paratyphoid, and malaria, and just under half of all cases of measles and chikungunya. While each included disease shows a different profile of traveller, this enhanced surveillance highlights diseases where VFR travel is most important.

Surveillance by reason for travel, country of birth and country of acquisition are key indicators for monitoring disease importation over time and for identifying changing disease epidemiology by destination and high-risk groups. Surveillance data are central to informing travel health messages and tailored pre-travel health advice. This enhanced surveillance of selected travel-associated diseases has demonstrated the value of collecting additional data routinely on travel and ethnic characteristics of notified cases. Expanded data collection to include at a minimum country of birth, parent's country of birth and reason for travel during routine follow-up of notified travel-associated disease is likely to improve our understanding of risk groups and inform the development of targeted disease control strategies, such as health promotion to high-risk communities. In Australia, the only national uniformly data collected on ethnicity is Indigenous status (i.e. Aboriginals and/or Torres Strait Islanders) [25]. States and Territories in Australia collect information on country of birth and language spoken at home; however, this is not uniformly collected and often incomplete, as seen in this study, with no data on country of birth for 18% of notified cases during the study period. Further, the absence of other indicators of ethnicity fails to adequately capture Australian-born children of immigrants, also at increased risk of travel-associated diseases, and representing 28% of cases and 45% of hepatitis A cases in this study. Defining a traveller as a VFR traveller by reason for travel alone has limitations and does not include an assessment of changes in the gradient of disease risk, as included in several definitions of a VFR traveller [26]. However, in the collection of routine data collections, such as immigration data (overseas arrivals and departures), and disease notification data, the collection of consistent and easy to collect data is important. Any ‘VFR travel’ in conjunction with country of birth and other indicators of ethnicity are likely to provide valuable data on risk groups without data collection issues. Several other studies have supported modifications to routine data collection of notifiable diseases to better understand immigrant health [25, 27, 28]. In Australia, and other countries with low or absent endemic disease, the index case of outbreaks of vaccine preventable diseases such as measles, typhoid and hepatitis A are most likely to be international travellers, and current travel medicine practices need to adapt to target health promotion to high-risk groups. Enhanced surveillance can inform who these high-risk groups are, as migrant patterns, travel patterns and global epidemiology change.

For the diseases included in this study, South Asia was the most common travel region and most common region of origin for immigrants and their Australian-born children, reflecting the global epidemiology of the included diseases and the large population of South Asian immigrants in Australia. One third of Australia's immigrant population are born in countries in Asia, including 9·4% from South Asia, with India the fourth most common country of birth after the UK, New Zealand and China [24]. India was disproportionately represented as the likely country of acquisition of cases, highlighting the potential benefit of more targeted public health primary prevention strategies to travellers to that region. Globally, South and Central Asia is the predominant region of travel for acquisition of enteric fevers [27, 29]. While acquisition of hepatitis A, hepatitis E and paratyphoid was predominantly during travel to South Asia, a high proportion of cases were reported after travel to South East Asia, reflecting the high volume of travel between Australia and the region. Measles cases originating from South East Asia during the study period reflects the large outbreak in the Philippines following Typhoon Haiyan [30]. Humanitarian emergencies commonly result in large disease outbreaks and VFR travellers to such destinations may be at increased risk. Our study indicated that VFR travel was an important factor in hepatitis E acquisition. There are no previous studies reporting hepatitis E acquisition by reason for travel in Australia, and case data from other developed countries is limited. This likely reflects the lower number of reported cases, with 13/1000 cases of hepatitis E reported from GeoSentinel sites compared to 40/1000 for hepatitis A [31]. However, locally acquired cases of hepatitis E may be under-ascertained as seroprevalence of hepatitis E antibodies in blood donors without as history of travel was 3·4% (compared to 6·4% of donors with a history of travel) [32]. Further, prior to mid-2014 locally acquired hepatitis E was not recognized in Australia as the case definition for notification included a requirement for a history of travel during the incubation period.

Just over half of malaria (54%) and 45% of chikungunya cases were VFR travellers. Australian notification data for these vector-borne diseases have not previously been reported by reason for travel, but other developed countries show a high proportion of malaria cases [1, 33, 34] in VFR travellers. While Papua New Guinea and South Asia remain the most common regions of acquisition of imported malaria in Australia [35], the large proportion of cases from sub-Saharan Africa and an increase in *Plasmodium falciparum* cases as a proportion of total imported malaria in Australia [17] and New Zealand [28] have previously been reported and reflect the changing patterns of migration and travel in the region. Early in the re-emergence of chikungunya in 2004, cases were predominantly returning tourist travellers to islands in the Indian Ocean; however, changes in the global epidemiology of this infection, including large ongoing outbreaks around the globe, has resulted in an expansion of the origins of imported disease to include India and countries in South East Asia [35–37], resulting in an increased risk for VFR travellers [38] of ethnicities common in Australia. The source countries of immigration and global disease outbreaks should inform travel health messages.

Children, either travelling with immigrant parents or immigrants themselves, were over-represented, particularly for vaccine-preventable diseases, and represented 35% of enhanced surveillance cases. Very little research focuses on paediatric VFR travellers, despite data indicating an increased risk of several travel-associated infections and a greater likelihood of VFR travel. Our data show cases with vaccine-preventable diseases were younger than those with non-vaccine-preventable diseases. Age and vaccination status are important considerations and may contribute to the high proportion of children in our study. Several studies have shown an inverse association between VFR travel and age [7, 39, 40] and indicate paediatric VFR travellers to be at increased risk of hepatitis A [41, 42] and typhoid [43], compared to children travelling for other purposes. The risk of travel-associated hepatitis A for British children aged <15 years in the early 1990s, prior to the availability of the hepatitis A vaccine, was 120/100 000 for VFR travellers compared to 15/100 000 for other travellers [41]. Pre-travel surveys of adult travellers show suboptimal vaccine uptake by VFR travellers [7, 8]. Despite the risk profile, little data are available on vaccine uptake of paediatric travellers with US travel clinic data indicating typhoid vaccine uptake was lower in English-speaking paediatric VFR travellers compared to travellers for other reasons [44]. In Australia, while some charges for a clinic visit may be incurred, vaccines on the National Immunization Program [45] are provided free of charge to age-eligible residents, which includes measles vaccine. However, typhoid and hepatitis A vaccines are not provided free of charge in Australia, except for hepatitis A vaccine for Indigenous children in high-risk jurisdictions [45]. Parental awareness of the need for pre-travel health assessment may be low in the immigrant population. In a study of Australian postpartum mothers originating from high tuberculosis prevalence countries, of those planning to return with their child to their country of birth, only 10% were aware of BCG vaccination recommendations [46]. Further, of immigrant participants, 24% were recent immigrants, arriving within the past 5 years. Recent immigrants are more likely to have barriers to accessing health services, including English-language proficiency [47] and may not be aware of the risks associated with VFR travel for themselves or their children [2]. These studies and our results highlight the need for targeted travel health education to VFR travellers of the need for vaccination, especially to parents of children who travel to VFR.

There are some limitations to the study. We excluded newly arrived visitors and immigrants from the enhanced surveillance, who comprised over 10% of the notified cases. While the aim was to capture travel characteristics of departing Australians, we may have missed important characteristics of cases of imported disease in visitor arrivals and disease in immigrants on arrival to Australia. Further, notified cases during the study period could opt out of the enhanced surveillance, with 35% of notified cases completing the enhanced surveillance. While there was no difference in the proportion of total and sample cases by age or gender, we may have underestimated the proportion of notified cases who were immigrants, with 18% of notified cases with an unknown country of birth. Response rates were also lower for malaria and chikungunya notifications due to the additional steps required to contact cases not routinely followed up by public health officers. Further, we included the selected travel-associated diseases due to their high likelihood of notification. However, they may not be representative of all travel-associated diseases. For example, dengue was not included in the study as the large number of notified cases (1842 in 2013 [48]) was beyond the scope of this prospective study. Increased notification of dengue in Australia has been linked to overseas travel to Bali, Indonesia, a popular tourist destination [49], but the proportion linked to VFR travel by Australians remains unknown. We reported travel by destination region and country which may not reflect the patterns of disease risk within countries. Our results may not be representative of all States and Territories in Australia. However, NSW and Victoria are the two largest States in Australia, with 57% of the estimated 2014 Australian population [18] and just over half (51%) of notified cases of the included diseases were notified in these States [48]. International airports in Melbourne, Victoria and Sydney, NSW account for 65% of Australian international passenger arrivals and departures [19]. Travel-associated disease outbreaks are therefore more likely to occur in these cities, such as the large outbreak of measles in South-west Sydney in 2012 [50].

Coupled with international travel, disparities in the prevalence of infectious diseases between low-burden countries such as Australia and high-burden countries, presents a challenge to national infectious disease control efforts. VFR travellers are a high-risk group for travel-related infectious diseases, and we have described specific risk groups and countries of acquisition in this study, which may assist in the development of more targeted primary prevention strategies. Disease prevention in this group of international travellers will have implications for the importation of infectious diseases into Australia. The expansion of national notification data to include routine collection of travel history, reason for travel as well as indicators of cultural and linguistic data including country of birth, parents’ country of birth and language spoken is important in monitoring high-risk groups in the importation of infectious diseases into Australia.
